# 216 Churchtown Walk & Talk Programme for Older Adults

**DOI:** 10.1093/eurpub/ckae114.009

**Published:** 2024-09-26

**Authors:** Shane Lee, Dearbhaile Oratis, Catherine Mulvihill, Claire Nicholl

**Affiliations:** Health Service Executive, Bray, Ireland; Health Service Executive, Bray, Ireland; Health Service Executive, Bray, Ireland; Health Service Executive, Bray, Ireland

## Abstract

**Purpose:**

Many community-based services discontinued due to the Covid-19 pandemic. Local primary care teams encountered clients exhibiting deconditioning, reduced muscle strength, diminished routines, and lacking confidence in movement. Loneliness and social isolation were increasingly evident.

**Project Description:**

Development: Churchtown Walk & Talk was developed, in partnership between the health service and Making Connections (befriending and wellbeing charity), to respond to local population’s needs.

Two walking groups were piloted (n = 22), aimed at addressing the observed muscle strength deterioration and social isolation. Eligible clients opted in following formal assessment by primary care physiotherapist/occupational therapist, and participants decided the route, distance and duration of the walk. On completion, participants were accompanied to a local café for further social engagement.

90% of pilot participants showed improved muscle strength. All participants reported a positive impact on their health and wellbeing.

Implementation: Following the success of the pilots, five additional groups (n = 62) were delivered, aimed at increasing:

• PA levels

• Muscle strength to reduce frailty

• Wellbeing through socialisation

**Evaluation:**

Pre/post outcomes were measured at weeks one and six, for five walking groups (n = 57).

Mean time score for Five Times Sit to Stand decreased (25.40s to 17.74s). Hand Dynamometer Grip Strength improved by 1.44kg (left hand) and 1.04kg (right hand). The mean Short Warwick Edinburgh Mental Wellbeing Scale score increased from 28.53 to 29.35.

77% of participants were engaging in PA for 5-7days post programme versus 58% pre intervention, and 90% of participants reported a positive impact on their health and wellbeing upon completion.

**Dissemination:**

Following the success of the programme, further adapted groups are planned in 2024 with expansion into other healthcare networks with an identified need.

**Conclusions:**

The population in Ireland is aging_1_, and frailty increases risk of disability; falls and cognitive decline. Interdisciplinary and multiagency collaboration for targeted local interventions may help to reduce risks for older adults. Meaningful engagement with participants allows tailored programmes which may improve engagement and outcomes.

**Reference:**

1. Sheehan, A. and O’Sullivan, R., (2020) Ageing and Public Health – an overview of key statistics in Ireland and Northern Ireland. Dublin: Institute of Public Health.

